# From respiratory infections to environmental exposures: CC16 is a key modulator of airway homeostasis

**DOI:** 10.3389/fmed.2026.1869095

**Published:** 2026-06-22

**Authors:** Kayleigh A. Berthiaume Fox, Julia Li, Riley D. Hellinger, Julie G. Ledford

**Affiliations:** 1 Department of Biomedical Engineering, College of Engineering, University of Arizona, Tucson, AZ, United States; 2 Medical Scientist Training Program, College of Medicine, University of Arizona, Tucson, AZ, United States; 3 Asthma and Airway Disease Research Center, University of Arizona, Tucson, AZ, United States; 4 Department of Physiology, College of Medicine, University of Arizona, Tucson, AZ, United States; 5 Department of Cellular and Molecular Medicine, College of Medicine, University of Arizona, Tucson, AZ, United States; 6 BIO5, University of Arizona, Tucson, AZ, United States

**Keywords:** anti-inflammatory, CC16, chronic lung disease, environmental exposures, respiratory infection, *SCGB1A1*, antimicrobial, club cell secretory protein

## Abstract

Club cell secretory protein 16 (CC16; *SCGB1A1*) is a multifunctional secretoglobin predominantly produced by nonciliated airway epithelial cells and is increasingly recognized as a central regulator of airway homeostasis. Beyond its long-standing role as a biomarker of epithelial integrity, accumulating clinical and experimental evidence highlights CC16 as an active modulator of inflammation, host defense, redox balance, and tissue remodeling. Reduced CC16 levels are a consistent feature of chronic inflammatory lung diseases, including asthma, chronic obstructive pulmonary disease, and cystic fibrosis, as well as following diverse environmental and occupational exposures. Alterations in CC16 dynamics are also observed during respiratory infections, where compartment-specific changes in CC16 concentrations reflect epithelial injury, barrier dysfunction, and disease severity. Mechanistically, CC16 exerts anti-inflammatory and antimicrobial effects through interactions with integrins, lipid mediators, and innate immune signaling pathways in both epithelial and immune cells, while also contributing to antioxidant defense and mitochondrial homeostasis. Environmental exposures, including tobacco smoke, air pollutants, and xenobiotics, profoundly influence CC16 expression in a context- and duration-dependent manner, whereas factors such as retinoids can restore CC16 production and epithelial repair. In this review, we synthesize current clinical, experimental, and mechanistic evidence to define the multifaceted roles of CC16 in airway biology, emphasize its relevance to susceptibility and outcomes in respiratory disease, and highlight critical knowledge gaps that must be addressed to advance CC16 as both a biomarker and therapeutic target.

## Introduction

Club cell secretory protein 16 (CC16), is a highly abundant anti-inflammatory and immunomodulatory protein secreted predominantly by club cells and nonciliated airway epithelial cells (1, 2). As a homodimeric pneumoprotein belonging to the secretoglobin family, CC16 occupies a unique position at the interface between epithelial biology and immune regulation. Reflecting its early discovery across multiple biological contexts, CC16 has been described in the literature under several names, including CCSP, CC10, human protein-1, urine protein-1, blastokinin (3), secretoglobin, and uteroglobin (4). Encoded by the *SCGB1A1* gene on chromosome 11q12.3–13.1 (5), CC16 was first identified unexpectedly in the urine of patients with renal failure (6), an observation that presaged its systemic detectability and biomarker potential. Subsequent studies have demonstrated that CC16 is broadly distributed beyond the lung, with measurable levels detected in the endometrium (7), amniotic fluid (8), breast milk (9), nasal lavage fluid (10–12), sputum (13), bronchoalveolar lavage fluid (BALF), and serum (14) (Table 1). Despite this widespread presence, the lung remains the primary site of CC16 synthesis, underscoring its importance as a marker of airway epithelial integrity and homeostasis. Indeed, circulating and airway CC16 levels are increasingly recognized as reflective of epithelial barrier function and epithelial-immune crosstalk within the respiratory track.

**Table 1:** Detection of CC16 in various compartments.

Source	Detection assay	Potential role in infection/disease	References
Club cells (terminal bronchioles, pulmonary edema fluid)	ELISA, IHC, IF, EM	CC16 is a marker of epithelial integrity and permeability. Decreased in smokers and patients with chronic obstructive pulmonary disease (COPD).	(1, 123)
Bronchoalveolar lavage fluid (BALF)	ELISA; automated latex immunoassay	BALF CC16 is predictive of diseases such as asthma and COPD and changes to epithelial barrier permeability.	(124–126)
Blood serum and plasma	ELISA; Latex immunoassay	Chronic heart failure and intake of uricosuric agents increased serum CC16. Obesity, arterial hypertension, and low gut microbiota diversity decreased serum CC16 levels. Serum CC16 levels are increased in patients with sarcoidosis, showing its potential as a marker of air-blood barrier damage. Serum CC16 levels are decreased in patients with asthma, COPD, cystic fibrosis (CF), etc.	(82, 127)
Umbilical cord blood	Automated latex immunoassay	Cord blood CC16 levels are positively associated with gestational age and negatively associated with the development of bronchopulmonary dysplasia (BPD) and respiratory distress syndrome (RDS).	(128, 129)
Urine	Trichloroacetic acid-Ponceau S method; MRM/MS; ELISA	There is a high correlation between urinary CC16 and serum CC16, making urinary CC16 a potential non-invasive biomarker alternative in the context of air pollution and lung permeability. Urinary CC16 increases shortly after exercise.	(6, 130, 131)
Cerebrospinal fluid (CSF)	ELISA immunosorbent assay	CC16 in the CSF originates from serum and is positively associated with age (higher in males than females).	(132)
Endometrium	RT-PCR, ELISA, IHC, Masterblot mRNA analysis	CC16 expression is increased during the luteal phase of the menstrual cycle and is upregulated by progesterone.	(4, 7)
Amniotic fluid	Immunoassay for CC16 detection in BAL; ELISA immunosorbent assay	CC16 in amniotic fluid is derived from fetal lung and may be a useful gestational marker of lung growth and development.	(8, 132)
Breast milk	Very low levels found (70 to 200 pg./mL), measured by ELISA	Increases in plasma CC16 is not due to uptake from breast milk.	(9)
Nasal lavage fluid	ELISA; Western blot	CC16 levels are decreased in children with birch pollen-induced allergic rhinitis. Levels of CC16 in nasal lavage fluid were inversely correlated with occurrence of metachromatic cells (MC, mast cells and basophils) in allergic inflammation.	(10–12)

ELISA = Sandwich enzyme-linked immunosorbent assay, IHC=Immunohistochemistry, IF=Immunofluorescence, EM = Electron microscopy, MRM = Multiple reaction monitoring, MS = Mass spectrometry, RT-PCR = Reverse transcription-polymerase chain reaction.

Notably, reduced CC16 levels are a consistent feature observed in chronic airway diseases, including asthma (15–17), COPD (18), and cystic fibrosis (19). Decreased CC16 concentrations are also seen in specific environmental exposure contexts, such as cigarette smoking (20) and occupational exposure among firefighters (21). These clinical and environmental settings share a common phenotype of heightened airway inflammation, epithelial injury and dysregulated immune responses. Intriguingly, individuals with the aforementioned chronic lung diseases or those with harmful inhalational exposures are also more susceptible to recurrent respiratory infections, raising the possibility that diminished CC16 levels may contribute directly to impaired host defense, which is plausible given the noted anti-inflammatory (22, 23) and anti-microbial (24, 25) properties attributed to CC16.

In this review, we synthesize current clinical and experimental evidence to examine the role of CC16 in lung health and disease, with a particular focus on respiratory infections and environmental exposures. Although many of CC16’s exact mechanisms remain unknown, we highlight emerging insights into CC16 as both a regulator of airway immune responses and a potential biomarker of epithelial dysfunction. Here, we aim to clarify its relevance in susceptibility to infection and chronic inflammatory lung diseases.

## CC16 in human respiratory infections—clinical investigations

Clinical studies in both pediatric and adult populations have demonstrated alterations in CC16 levels during viral respiratory infections. Notably, certain viral infections are associated with increased CC16 levels, whereas others are associated with decreased levels. Consequently, the utility of CC16 as a biomarker of disease severity, epithelial injury, and clinical outcomes likely depends on both the type of viral infection and the age of the patient.

In children with adenovirus (ADV) pneumonia, Yuan et al. identified low serum CC16 levels as an independent risk factor for severe disease (26). In contrast, infants hospitalized with acute bronchiolitis, most commonly caused by Respiratory Syncytial Virus (RSV), serum CC16 was significantly elevated compared to healthy controls (27). Other studies by Egron et al. reported that urinary CC16 levels increase proportionally with disease severity in infants hospitalized with acute bronchiolitis, suggesting its additional utility as a noninvasive urine biomarker (28).

Serum CC16 has been extensively evaluated as a prognostic biomarker for disease progression, hospital length of stay, and mortality in viral-associated acute lung injury. In adults with H1N1- or SARS-CoV-2-associated acute lung injury (ALI) or acute respiratory distress syndrome (ARDS), Moore et al. reported strong correlations between CC16 levels and hospital length of stay (28). Similarly, Tiezzi et al. demonstrated that higher serum CC16 levels were associated with increased mortality risk in patients with SARS-CoV-2-induced ARDS (29). Consistent with these findings, Rohmann et al. observed elevated serum CC16 levels in SARS-CoV-2 infected patients compared to healthy controls. Kardol-Hoefnagel et al. further demonstrated serum CC16 levels predict COVID-19 severity, with elevations preceding ICU admission and reaching the highest levels in non-survivors (29).

In contrast, Yin et al. reported reduced serum CC16 levels, decreased numbers of CC16^+^ epithelial cells, and lower pulmonary CC16 mRNA expression in severe COVID-19 cases (30). Reduced circulating CC16 levels have also been described in community-acquired pneumonia (CAP) (31). In adults hospitalized with bacterial- or viral-associated CAP, Li et al. reported that serum CC16 levels declined with disease progression; however, lower levels on admission were independently associated with an increased risk of poor in-hospital outcomes. Notably, Kurowski et al. found that elite athletes exhibited lower serum CC16 levels compared with healthy controls, which correlated with an increased susceptibility to frequent respiratory tract infections, including parainfluenza, RSV, and adenovirus (32).

Growing evidence further highlights a critical role for CC16 in host defense against bacterial respiratory pathogens, as well. A human population-based study by Dy et al. demonstrated that CC16 is protective against *Mycoplasma pneumoniae* (*Mp*) infection, especially in patients with asthma (33). Individuals with serologic evidence of *Mp* infection and low serum CC16 levels exhibit greater impairments in lung function, suggesting CC16 may mitigate infection-associated airway dysfunction (33).

Taken together, clinical findings demonstrate that CC16 levels are likely influenced by the type of respiratory infection and vary across various biological compartments such as serum, BALF, and urine. CC16 levels detected during or after respiratory infection likely reflects alterations in airway epithelial integrity, permeability, and inflammatory status. More recent studies suggest that CC16 may serve not only as a biomarker of injury, but actively contribute to host defense, particularly in the context of respiratory infections.

## Insights into CC16 function from experimental animal models

Animal models have been essential for elucidating the regulatory and protective functions of CC16 in the lung. In particular, two well-characterized CC16 gene-deficient knock out (KO) mouse strains have provided foundational mechanistic insights into CC16 biological function (34, 35). Studies across these models demonstrate that CC16 deficiency disrupts airway physiology, promotes structural remodeling, and enhances inflammation.

Zhai et al. demonstrated that, under baseline conditions, CC16-deficient mice exhibit significantly elevated total airway resistance compared to WT mice (36). CC16-deficiency was also associated with increased tissue elastance and greater viscoelastic energy dissipation, consistent with a stiffer and more heterogenous lung parenchyma (37). These mechanical abnormalities were further exacerbated following methacholine challenge, during which CC16-deficient mice displayed pronounced airway hyperreactivity and greater increases in airway resistance relative to CC16-sufficient controls.

Laucho-Contreras et al. reported that unchallenged CC16-deficient mice developed significant distal airspace enlargement and increased lung compliance between 6- and 18-months of age, reflecting reduced elastic recoil and an emphysema-like pathology (38). Histopathological analyses revealed peripheral airspace enlargement accompanied by small airway fibrosis, characterized by thickened basement membranes, smooth muscle hypertrophy, and increased peribronchiolar collagen deposition. These structural alterations correlated with elevated expression of key remodeling genes such as *Col1A1, Col3A,* and *α-SMA*, which is indicative of a transcriptional program favoring irreversible airway remodeling and disease progression (39). Collectively, these findings underscore a critical role for CC16 in preserving normal airway architecture and maintaining lung function, with implications for obstructive lung diseases, including asthma and COPD.

## CC16’s mechanisms of action

### Anti-inflammatory mechanisms

CC16 mediates its anti-inflammatory effects through both receptor-mediated and intracellular signaling mechanisms (Table 2). Notably, CC16 directly binds to the lipoxin A4 receptor ALX/FPR2 (formyl-peptide receptor 2), a GPCR highly expressed on neutrophils, monocytes, and macrophages (40). Reduced ALX/FPR2 expression has been implicated in the pathogenesis of asthma, underscoring the relevance of this pathway (41, 42). Engagement of ALX/FPR2 by CC16 inhibits serum amyloid A (SAA)-induced phospholipase A_2_ (PLA_2_) activity (43), thereby limiting key downstream lipid-mediated inflammatory signaling (44). Consistent with this mechanism, CC16 suppresses SAA-dependent IL-8 production in human endometrial cells and inhibits chemotaxis in promyelocytic leukemia cells (43).

**Table 2:** Binding partners of CC16.

Binding partner	Location	CC16’s biological effects	Reference
Blastocysts	Extracellular	Stimulates expansion and continued growth of blastocyst during embryonic development	(133)
Calcium	Intracellular	Inhibits phospholipase A2 (PLA2) by sequestering calcium ions	(134)
Cubilin	Intracellular	Uptakes cubulin and transport to lysosomes for degradation	(135)
Fibronectin (Fn)	Extracellular	Forms Fn-UG heteromers to prevent fibronectin self-aggregation	(136, 137)
FPR2 (ALX)	Plasma membrane	Downregulates SOCS-3 and STAT-1 to suppress T-helper 2 (T(H)2) cell differentiation	(59)
HSP60	Intracellular	Suppresses nuclear factor κB (NF-κB) signaling and subsequent inflammatory response	(75)
LIMR (LMBR1L)	Extracellular	Suppresses migration and invasion of cancer cells expressing LIMR	(138)
Progesterone, estradiol-17beta, testosterone, etc.	Circulation	Acts as transport protein for various steroid hormones	(139)
methylsulfonyl-polychloro-biphenyl (PCBs) derivatives	Extracellular	Acts as transport protein for PCBs in lung tissue and circulation	(140)
Microsomes	Intracellular	Mediates physiological effect on cell membranes, specific downstream signaling not discussed in detail	(141)
PGD2	Intracellular	Inhibits tyrosine phosphorylation of p38 mitogen-activated protein kinase (MAPK) and p44/42 MAPK; Blocks NF-κB activation and subsequent inflammatory response	(22)
Phospholipid liposomes	Intracellular	Regulates phospholipid homeostasis within airways and/or secretory pathways of club cells	(142)
Phosphatidylcholine and phosphatidylinositol	Intracellular	Sequesters molecules in hydrophobic cavity and acts as carrier protein	(143)
Platelets	Circulation	Triggers actin polymerization in resting platelets and inhibits their aggregation	(144)
Progesterone	Circulation	Localizes hormone in uterine epithelium and catalyzes its metabolism	(145)
Retinoids	Circulation	Acts as transport protein, specific biological effects not discussed in detail	(146)
VLA-2 (*α*2*β*1)	Plasma membrane	Upregulates SPLUNC1, drives antimicrobial host defense, reduces *Mycoplasma pneumoniae* (*Mp*) pathogen burden	(25)
VLA-4 (*α4β*1)	Plasma membrane	Triggers various anti-inflammatory and protective mechanisms against *Mp* infection	(52)

UG = uteroglobin, FPR2/ALX = N-formyl peptide receptor 2/Lipoxin A4, HSP60 = Heat Shock Protein 60, LIMR/LMBR1L = Lipocalin-1 Interacting Membrane Receptor/Limb Development Membrane Protein 1-Like, PGD2 = Prostaglandin D2, VLA-2 = Very Late Antigen 2, VLA-4 = Very Late Antigen 4.

Beyond antagonizing SAA, CC16 also suppresses prostaglandin synthesis though inhibition of the PLA2-arachidonic acid (AA) pathway (45). In experimental models of allergic inflammation, OVA-challenged CC16-deficient mice exhibit significantly increased prostaglandin D2 (PGD2) levels in BALF and elevated pulmonary COX-2 gene expression (22). Pre-treatment with recombinant CC16 markedly reduces PGD2 production and COX-2 expression following OVA challenge. Given that PGD2 is a potent eosinophil chemoattractant and that COX-2 drives the generation of proinflammatory lipid mediators, both of which are upregulated in allergic asthma, these findings underscore a central role for CC16 in modulating lipid-mediated inflammatory pathways in asthma (46, 47).

Recombinant CC16 also reduces the expression of pro-inflammatory cytokines, including TNF-*α*, IL-6, and IL-8, at both the transcript and protein levels in an *in vivo* model of COPD induced by long-term smoke exposure (48). These findings are consistent with prior studies demonstrating that CC16 attenuates NF-kB signaling in airway epithelial cells and macrophages (49, 50). Classical NF-kB activation is facilitated by a wide range of stimuli, including pro-inflammatory cytokines, microbes, and oxidative stress. We propose that CC16 is likely to modulate NF-ĸB through several proposed mechanisms. One, through binding of extracellular inflammatory ligands, leading to less receptor stimulation, such as TNFR, TLRs, or IL-1R, and subsequent downstream signaling (Table 2). CC16 has also been shown to inhibit NF-ĸB translocation, likely through intracellular suppression of IĸB -*α* activation (51). Furthermore, CC16 binds to VLA-4, likely competing with ligands such as VCAM-1 or fibronectin, preventing subsequent nuclear NF-ĸB translocation (52).

In the context of exposure to diesel exhaust particles (DEP), an inverse relationship has been reported between CC16 levels and pro-inflammatory cytokine expression in human airway epithelial cells (53). Notably, DEP exposure transiently increases TNF-*α* production, which in turn appears to stimulate CC16 secretion as part of an anti-inflammatory feedback response. The subsequent increase in CC16 then suppresses further TNF-α production, thereby limiting DEP-induced inflammatory signaling (53).

IFN-*γ* has also been shown to increase CC16 mRNA levels both *in vivo* (54) and *in vitro* (55), while TNF-α increases CC16 secretion in human bronchial epithelial cells *in vitro* (56). Increases in CC16 mRNA are thought to be controlled, at least in part, by post transcriptional regulation, whereby mRNA stability is prolonged by RNA binding proteins (57). Together, these findings implicate CC16’s participation in a cytokine-inducible, anti-inflammatory feedback loop within the airway epithelium.

### Interaction with leukocytes

In addition to action on the airway epithelium, CC16 also modulates inflammatory pathways in leukocytes, which impacts both their recruitment and activation. In a *Pseudomonas aeruginosa*-derived LPS model, CC16 deficiency leads to increased F4/80 + macrophages in BALF and an increased expression of TLR4 (a pattern recognition receptor of LPS) on those macrophages, suggesting an increase in LPS-responsiveness in the absence of CC16 (58). Recombinant CC16 (rCC16) has been generated by several groups as an experimental augmentation therapy for models of CC16-deficency. rCC16 is usually produced by a bacterial expression system, where the gene is cloned into a plasmid and transformed, often into *Escherichia coli*, and subsequently purified. rCC16 treatment of macrophages during LPS challenge reduced NF-κB transcriptional activity through MAPK inhibition (50). In dendritic cells, CC16 suppressed STAT1 signaling and downregulated SOCS-3, which decreased both their migration and T_H_2-associated cytokine production (59). CC16 competes with SAA for binding to FPR2 on T_H_2 cells, further supporting its mechanistic role in controlling inflammatory signaling.

**Figure 1:**
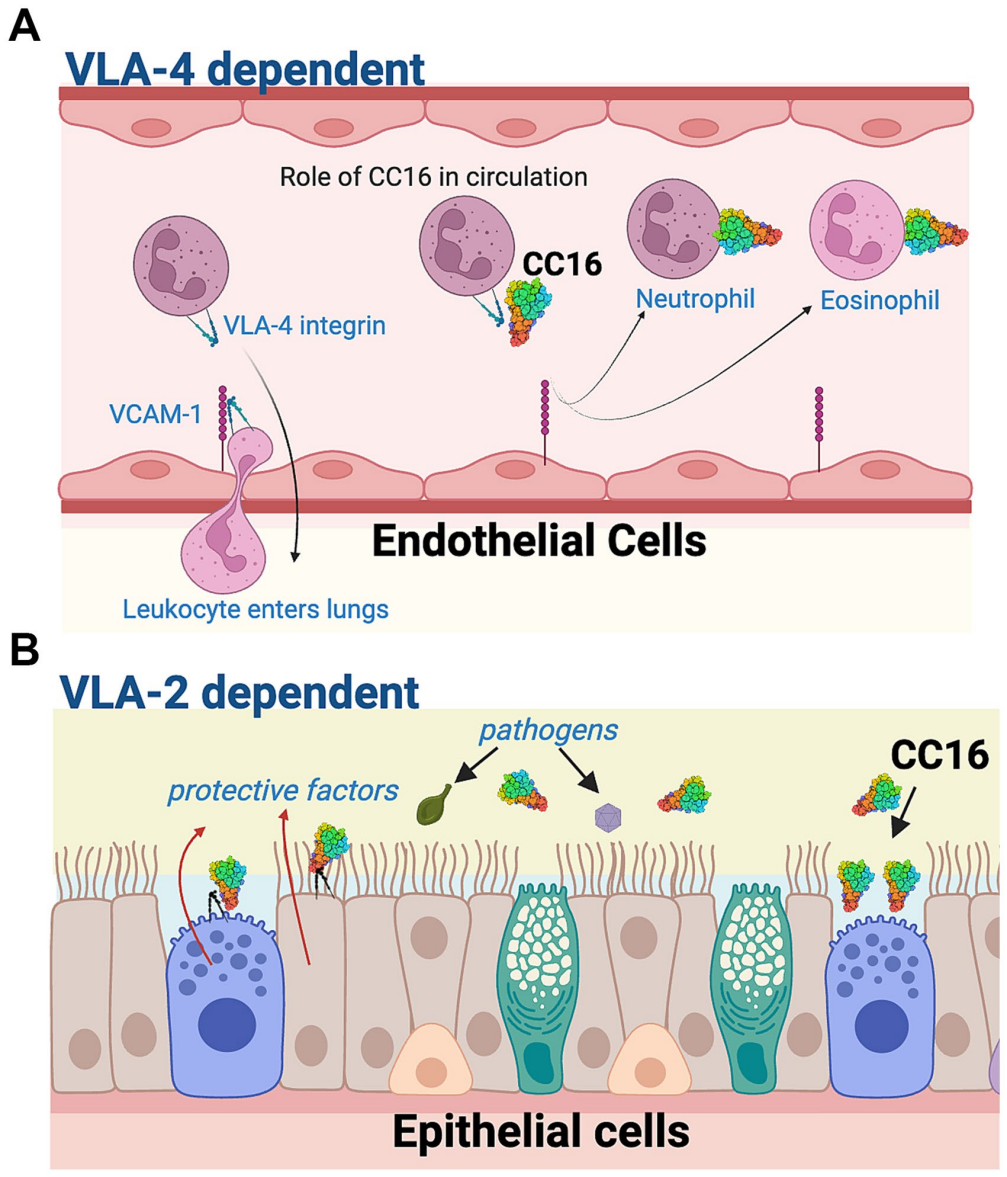
Proposed mechanisms of CC16 interactions with integrins. **(A)** CC16 binds Very Late Antigen-2 (VLA-2, integrin *α*2*β*1), expressed on the surface of epithelial cells in the airway, leading to increased production of anti-microbial factors. **(B)** CC16 binds VLA-4 (integrin *α4β*1) on the surface of circulating leukocytes, leading to decreased immune cell infiltration to sites of inflammation.

CC16 is also a binding partner of very late antigen VLA-4 (α_4_β_1_) (52). VLA-4 is expressed on the cell surface of leukocytes and binds to VCAM-1, mediating immune cell adhesion and extravasation (60) (Figure 1B). Johnson et al. discovered that CC16 binds VLA-4, via its leucine-valine-aspartic acid (LVD) integrin-binding motif, on activated leukocytes and reduces their ability to bind to endothelial cells and extravasate into the lung from circulation. rCC16 with mutations to the LVD site render it is less functional in reducing leukocyte adhesion to endothelial cells which results in more neutrophil recruitment in BALF of *Mp*-infected mice compared to those receiving rCC16 alone (52).

An expanding body of evidence also supports a role for CC16 in the regulation of neutrophil responses. In CC16 knock-out (KO) mice, adenoviral infection results in increased neutrophil infiltration and elevated expression of proinflammatory cytokines, including IL-6, IL-1β, and TNF-*α*, as well as neutrophil-attracting chemokines, MIP-1α and MIP-2 (61). Similarly, CC16 KO mice infected with *Pseudomonas aeruginosa* exhibit enhanced neutrophil accumulation in the lungs and increased levels of IL-1β and TNF-α compared to CC16-sufficient controls (62). In *Mp-*infected models, CC16-deficient mice demonstrate increased neutrophil trafficking to the alveoli, airways, and circulation relative to CC16-suficient controls (52). Loss of CC16 is also associated with neutrophilia and eosinophilia in mice challenged in the OVA model of allergic airway disease (63).

Taken together, CC16 likely impacts neutrophil recruitment by two distinct mechanisms. First, by binding to VLA-4 on activated neutrophils in circulation, CC16 limits their transmigration into the lungs (52). Second, by downregulating NF-kB and p38 MAPK pathways, CC16 inhibits the production of factors such as IL-8, IL-6 and TNF-*α*, which would result in less neutrophil recruitment (50).

### Antimicrobial functions

Iannuzo et al. discovered that CC16-deficient primary mouse tracheal epithelial cells (MTECs) have decreased secretion of several key antimicrobial and antiviral proteins, including SPLUNC1, surfactant protein D (SFTPD), surfactant protein B (PSPB), lactotransferrin (TRFL), lysozyme C-2 (LYZ2), and secretoglobin family 3A member 2 (SCG3A2). Further, mRNA expression of *SCGB1A1* and *BPIFA1* from bronchial epithelial cells collected as part of the Severe Asthma Research Program (SARP) demonstrated significant positive correlation. Further meta-analysis from this study also revealed *BPIFA1, TF, LTF, SCGB3A2, SFTPD,* and *LYZ* to be positively and significantly correlated with CC16 expression (25). Induction of *SPLUNC1* secretion by CC16 was dependent on signaling via another integrin complex, VLA-2. VLA-2 consists of the integrins α_2_ and β_1_, and is highly expressed on cells lining the human airway epithelium (Figure 1A). Splunc1, as well as many of the other antimicrobial and antiviral factors associated with CC16 as described above (25), are under the control of the AP-1 promoter. This suggests that CC16 signals through the heterodimeric integrin VLA-2 complex on airway epithelial cells to induce AP-1 gene transcription of host defense factors, which would result in an enhanced response to respiratory pathogens.

Collectively, these findings highlight CC16 as a key regulator of antimicrobial defense, acting in part through interactions with integrins expressed on the surface of epithelial cells. Given that CC16 levels are reduced in individuals with asthma, COPD, and cystic fibrosis, and that antimicrobial defense factors similarly decline in the context of low CC16 expression, it is likely that diminished CC16 contributes to impaired host defense mechanisms in these chronic lung diseases and contribute to enhanced infection rates and exacerbations.

### Redox homeostasis

CC16 also plays a key role in maintaining redox homeostasis in the airway epithelium, a function closely linked to its anti-inflammatory effects. In response to ozone exposure, CC16-deficient mice exhibit higher levels of IL-6 and metallothionein (MT), an antioxidative protein (64), compared to CC16-sufficient mice (65). Notably, MT expression peaks at 8 h in CC16-sufficient mice but occurs 4 h earlier in CC16-deficient mice, suggesting a more rapid and pronounced oxidative stress response in the absence of CC16.

CC16’s antioxidative effects have also been studied in the context of PM2.5 exposure, a pollutant of atmospheric fine particulate matter that can reach the alveoli and lead to oxidative stress. Wang et al. showed that exposure to PM2.5 significantly decreased levels of glutathione (GSH) in murine lung epithelial cells, while increasing levels of reactive oxygen species (ROS) and malondialdehyde (MDA), an oxidative stress marker and end product of lipid peroxidation (66, 67). In this model, treatment with rCC16 increased expression of nuclear factor erythroid 2-related factor (Nrf2), a key cellular regulator against oxidative stress (68), and its downstream anti-oxidant enzyme, glutathione peroxidase 4 (GPX4). CC16 also decreased levels of MDA, intracellular ROS, and ACSL4, a catalyst of ferroptosis (69).

PM2.5 induces pyroptosis, a form of cell death characterized by the secretion of pro-inflammatory cytokines and oxidative stress (70, 71). Lin et al. found that PM2.5 exposed, OVA-sensitized asthmatic mice treated with nebulized rCC16 had reduced levels of pyroptotic and pro-inflammatory signaling factors such as TLR4, phosphorylated NF-κB, caspase-1, and IL-1β compared to asthmatic mice receiving vehicle (72). The authors also found that PM2.5 exposed human bronchial epithelial (BEAS-2B) cells treated with rCC16 had reduced pyroptosis and inflammation, similarly characterized by reduction in the expression of caspase-3, caspase-1, gasdermin D, HMGB1, and IL-1β compared to those receiving vehicle control (72).

Human bronchial epithelial cells exposed to cigarette smoke extract (CSE) had decreased levels of intracellular and mitochondrial ROS following treatment with rCC16 (73). Additionally, the levels of antioxidant enzymes involved in ROS scavenging in the mitochondria, including SOD1, SOD2, CAT, and GPX-4, were restored to their normal levels after treatment with rCC16. In line with this, Berthiaume Fox et al. reported enriched pathways related to antioxidant functions and glutathione metabolism in primary mouse tracheal epithelial cells (MTECs) treated with recombinant CC16 (74). Interestingly, this study also reported decreases in mitochondrial bioenergetics in CC16-deficient MTECs, which was rescued to baseline WT levels with rCC16 treatment.

CC16 is likely to regulate ROS via several mechanisms including preventing HSP60 activated NF-kB signaling (75). Additionally, overexpression of CC16 in BEAS-2B cells led to reduced cellular and mitochondrial ROS generation compared to BEAS-2B cells without overexpression (76). Their findings suggest that mechanistically, intracellular endocytosed CC16 may be providing protection against ROS generation during respiratory infections, while other components of the pathway are unknown. Taken together these data imply that CC16 is protective against oxidative stress and potentially, mitochondrial dysregulation, and that loss of CC16 may lead to an aberrant accumulation of ROS. At low levels, ROS direct critical signaling cascades, however, increased ROS can lead to dysregulation of immune cells, (77–79) and may subsequently increase susceptibility to infection.

## Environmental exposures and the impact on CC16 levels

Environmental exposures affecting CC16 levels can be characterized based on their impact to lung and club cell homeostatic functions.

### Acute epithelial injury and permeability response

Increases in serum CC16 levels are hypothesized to be due, in part, to acute epithelial injury and hyperpermeability. Acute exposures to cigarette smoke (80), and various xenobiotics like asbestos (81, 82), cadmium (83), and grain dust (84), may increase permeability between the lung-blood barrier to allow the small, 16 kDa molecule to translocate from the luminal compartment of the airways into the circulation.

Indeed, evidence supporting this transient increase in serum CC16 has been documented in firefighters immediately after responding to a fire (21, 85), with one study identifying CC16 levels returning to baseline 10 days later (85). In line with this, healthy volunteers with a single exposure to woodsmoke demonstrated increased serum CC16 four hours after the exposure, returning to baseline by the next day (86). Likewise, studies evaluating acute changes in environmental air pollution, such as exposure to PM2.5 and ozone exposure, have identified increased circulating CC16 concentrations (87, 88).

### Direct club cell loss or dysfunction

Decreased circulating CC16 can be attributed to direct club cell injury or loss due to toxic inhalation exposures, like tobacco smoke, toxicant-heavy metals, and pollutants. Particularly, club cells and CC16 have been studied extensively in the context of tobacco smoke exposure in both humans and animal models. Starting in the 1980’s, studies have shown that chronic exposure to cigarette smoke results in a decreased proportion of club cells throughout the airways (89). In line with this, clinical studies in humans have consistently reported decreased CC16 levels in smokers across multiple compartments including, BALF (14, 90), lung tissue (90, 91), and serum (14, 90, 92), attributed to the loss of club cell numbers.

Although loss of club cells is likely a major contributor to reduced levels of CC16 (5, 93, 94), additional mechanisms of epithelial dysfunction are likely involved (95). These include epigenetic silencing (96), decreased activation of pathways inducing endogenous CC16 production (97), or impairment of other non-ciliated cells in the airways that produce CC16 (98–100). Interestingly, some studies have also found sex and age differences in CC16 levels (79, 98–100).

Several other toxicants have been shown to facilitate loss of CC16 in both human and animal models, including inhaled silica (92, 101), sulfur dioxide (94), nitrogen oxides (102, 103), other inhaled toxicants (104–106), and several metals (83, 107, 108). Additionally, *in vivo* and *in vitro* models have demonstrated reduced CC16 levels after exposure to xenobiotics such as diesel engine exhaust (53, 109, 110), carpet dust (111), ovalbumin (112), and lung toxicants such as methylcyclopentadienyl manganese tricarbonyl (MMT), 4-Ipomeanol, alpha-naphtylthiourea, and paraquat (113, 114).

### Exposure dynamics

CC16 levels appear to have a time- and dose-dependent relationship with tobacco smoke, woodsmoke, and other noxious inhaled toxicants.

For instance, people with longer smoking histories and those who progress to more severe stages of COPD are correlated with the lowest CC16 levels (14, 115). When adjusting for cumulative smoke exposure (pack-years), those who are current smokers have lower CC16 levels than those who are former smokers (100). Laboratory models of chronic tobacco smoke-induced COPD have shown similar trends. Zhu et al. found decreased tissue expression of CC16 in chronically exposed non-human primates and mice compared to animals exposed only to filtered air (95). Similarly, Laucho-Contreras and colleagues reported that mice exposed to cigarette smoke for 6 months exhibited the greatest reduction in tissue CC16 expression compared to mice exposed for 1 or 3 months, relative to unexposed controls (115). Additional studies demonstrated that treatment of smoke-exposed mice with rCC16 attenuated pathological features seen in CC16-deficient animals, including emphysematous changes consistent with COPD (48, 116). Interestingly, *in vitro* bronchial epithelial cell culture models demonstrate decreased CC16 production with cigarette smoke extract exposure which returns to baseline after removal of the exposure, suggesting a temporal response of CC16-producing cells to noxious exposures (117).

While chronic exposure to tobacco smoke and the development of COPD are both risk factors for low circulating CC16, extended exposure to aerosolized commercial insecticide and air pollution have also been reported to decrease CC16 levels in a dose-dependent manner (118, 119).

### Retinoid-driven upregulation of CC16

Retinoids, including vitamin A and its active metabolite, all-trans retinoic acid (RA), are important regulators of CC16 expression in airway epithelial cells (120). RA has been shown to directly stimulate *SCGB1A1* transcription through retinoic acid receptor (RAR)– and retinoid X receptor (RXR)–dependent mechanisms, promoting club cell differentiation and maintenance of the secretory epithelial phenotype (121, 122). *In vivo* and *in vitro* studies demonstrate that vitamin A deficiency reduces CC16 expression, whereas retinoid supplementation restores CC16 levels and enhances club cell function, particularly during airway injury and repair (121). These findings position retinoid signaling as a key upstream pathway controlling CC16 expression and airway epithelial homeostasis.

Taken together, CC16’s demonstrates a biphasic response to inhaled environmental exposures, depending on exposure type, dose, and chronicity. This dynamic response highlights CC16 as a marker of acute airway inflammation, permeability, and chronic epithelial dysfunction.

## Conclusion

Current evidence establishes CC16 as far more than a passive marker of airway epithelial injury, positioning it instead as a contributor to immune modulation, antimicrobial defense, oxidative stress responses, and airway structural integrity. Clinical studies across diverse respiratory infections and chronic lung diseases demonstrate that alterations in CC16 levels closely track with disease severity, epithelial barrier dysfunction, and clinical outcomes, while experimental models reveal a causal role for CC16 in constraining inflammation, leukocyte recruitment, and tissue remodeling. Furthermore, environmental exposures emerge as powerful modifiers of CC16 expression, with acute increases often reflecting epithelial permeability and chronic reductions indicating impaired epithelial resilience and repair capacity. Importantly, emerging data identifying retinoids and other signaling pathways that regulate CC16 expression suggest actionable upstream mechanisms for restoring CC16 in disease settings. Future studies integrating longitudinal human cohorts, refined compartment-specific measurements, and targeted mechanistic models will be essential to disentangle context-dependent changes in CC16 and to define its potential in further understanding and treating complicated airway pathology.
